# Diagnostic Performance of ⁶⁸Ga-FAPI PET/CT, ¹⁸F-FDG PET/CT, and Breast MRI for Assessing Tumor Extent and Axillary Metastasis in Breast Cancer: A Histopathology-Referenced Retrospective Study

**DOI:** 10.1007/s00259-026-07953-9

**Published:** 2026-06-16

**Authors:** Deniz Esin Tekcan Sanli, Umut Elboga, Enes Yerdes, Ahmet Necati Sanli

**Affiliations:** 1 Department of Radiology, Faculty of Medicine, Gaziantep University, Gaziantep, Turkey; 2 Department of Nuclear Medicine, Faculty of Medicine, Gaziantep University, Gaziantep, Turkey; 3 Department of General Surgery, Abdulkadir Yuksel State Hospital, Gaziantep, Turkey

**Keywords:** Breast Cancer, 68Ga-FAPI PET/CT, 18F-FDG PET/CT, Magnetic Resonance Imaging, Axillary Metastasis, Multifocality

## Abstract

**Purpose:**

To compare the diagnostic performance of ⁶⁸Ga-FAPI PET/CT, ¹⁸F-FDG PET/CT, and magnetic resonance imaging (MRI) in assessing primary tumor size, detecting additional intramammary foci, and identifying axillary lymph node metastasis (ALNM) in patients with invasive breast cancer (IBC) undergoing upfront surgery without neoadjuvant chemotherapy (NAC), using postoperative histopathology as the reference standard.

**Methods:**

This retrospective study included 60 IBC patients who underwent total mastectomy without NAC. All patients had preoperative ⁶⁸Ga-FAPI PET/CT, ¹⁸F-FDG PET/CT, and breast MRI. Primary tumor size, multifocality/multicentricity (MF/MC), and ALNM were evaluated for each modality. FAPI and FDG were additionally assessed for extra-axillary lymph nodes, internal mammary nodes, and distant metastases. Agreement between imaging- and pathology-based tumor size measurements, as well as the diagnostic performance for MF/MC and ALNM, were evaluated using correlation analyses and standard diagnostic accuracy metrics.

**Results:**

MRI (ρ = 0.866) and ⁶⁸Ga-FAPI PET/CT (ρ = 0.844) demonstrated the strongest correlation with pathologic tumor size, whereas ¹⁸F-FDG PET/CT showed lower agreement (ρ = 0.780). Breast MRI (45.0%) and ⁶⁸Ga-FAPI-PET/CT (38.3%) were significantly superior to ¹⁸F-FDG PET/CT (18.3%) in detecting multifocal or multicentric disease (*p* < 0.001).

For ALNM detection, breast MRI achieved the highest sensitivity (87.5%) but showed lower specificity (72.7%). In contrast, ⁶⁸Ga-FAPI PET/CT combined high sensitivity (81.2%) with perfect specificity (100.0%), outperforming ¹⁸F-FDG PET/CT, which demonstrated lower sensitivity (43.8%) despite high specificity (93.2%).

Additionally, ⁶⁸Ga-FAPIPET/CT identified metastatic findings not detected by ¹⁸F-FDG PET/CT, including seven axillary lymph nodes (11.7%), 29 additional intramammary tumor foci (48.3%), and four distant metastases (6.7%).

**Conclusion:**

⁶⁸Ga-FAPI PET/CT demonstrated higher diagnostic performance than ¹⁸F-FDG PET/CT and accuracy comparable to breast MRI for primary tumor size estimation and detection of multifocal or multicentric disease. FAPI-PET/CT also provided higher specificity than MRI for ALNM assessment and identified additional metastatic lesions not detected by FDG-PET/CT, supporting its potential role as a complementary modality to MRI and an alternative functional imaging technique in selected patients.

## Introduction

Breast cancer is the most common malignancy among women, and accurate staging together with reliable assessment of the true extent of disease remains essential for optimizing both surgical and systemic treatment strategies [1]. Precise measurement of primary tumor size, identification of multifocal or multicentric disease, and correct evaluation of axillary lymph node status are pivotal components that directly influence surgical planning and overall clinical management [2, 3].

Breast magnetic resonance imaging (MRI) is a cornerstone modality in this context, owing to its excellent soft-tissue contrast and high sensitivity, particularly for detecting multifocal and multicentric involvement [2–4]. Nevertheless, several clinical studies have reported that, despite its high sensitivity, MRI does not always show perfect concordance with histopathology regarding the exact tumor size or the true extent of disease [4–6]. These discrepancies may arise from both biological and technical factors, including histologic subtype, molecular profile, stromal composition, tumor grade, background parenchymal enhancement, menstrual cycle phase, or acquisition-related variables [2, 7–10]. Furthermore, the limited specificity of MRI can lead to false-positive interpretations of benign lesions, potentially resulting in unnecessarily extensive surgery [11–13].

¹⁸F-FDG PET/CT provides functional assessment of tumor glucose metabolism; however, tumors with low proliferative activity, well-differentiated histology, or invasive lobular carcinoma (ILC) frequently demonstrate low FDG uptake [14, 15]. This may lead to underestimation of primary tumor size, missed multifocal or multicentric disease, or overlooked axillary metastases [14–18]. Therefore, neither high-resolution anatomical imaging nor glucose metabolism–based functional imaging alone may be sufficient to comprehensively characterize the true extent of breast cancer.

More recently, fibroblast activation protein (FAP) inhibitors have enabled FAPI-PET imaging, which targets activated stromal fibroblasts within the tumor microenvironment, producing high tumor-to-background contrast [19–21]. Because FAPI uptake is prominent even in tumors with low metabolic activity but rich stromal components, it may offer a complementary advantage in situations where FDG is limited [19–25]. Despite these promising characteristics, comparative studies directly evaluating FAPI-PET against both MRI and FDG-PET remain limited in the breast cancer literature.

Accordingly, this study systematically compared ⁶⁸Ga-FAPI PET/CT, ¹⁸F-FDG PET/CT, and breast MRI in women with invasive breast cancer undergoing upfront surgery without neoadjuvant chemotherapy. Primary tumor size, multifocality/multicentricity, and axillary lymph node metastasis were evaluated using postoperative histopathology as the reference standard. In addition, the ability of both PET tracers to detect extra-axillary lymph node involvement and distant metastases was assessed.

## Methods

### Patient Population

This retrospective study included women who underwent upfront total mastectomy between January 2023 and June 2025 at our institution, either due to multifocal/multicentric (MF/MC) disease suspected on preoperative imaging or patient preference, with a histopathologically confirmed diagnosis of primary invasive breast cancer and no prior neoadjuvant chemotherapy (NAC).

**Fig. 1:**
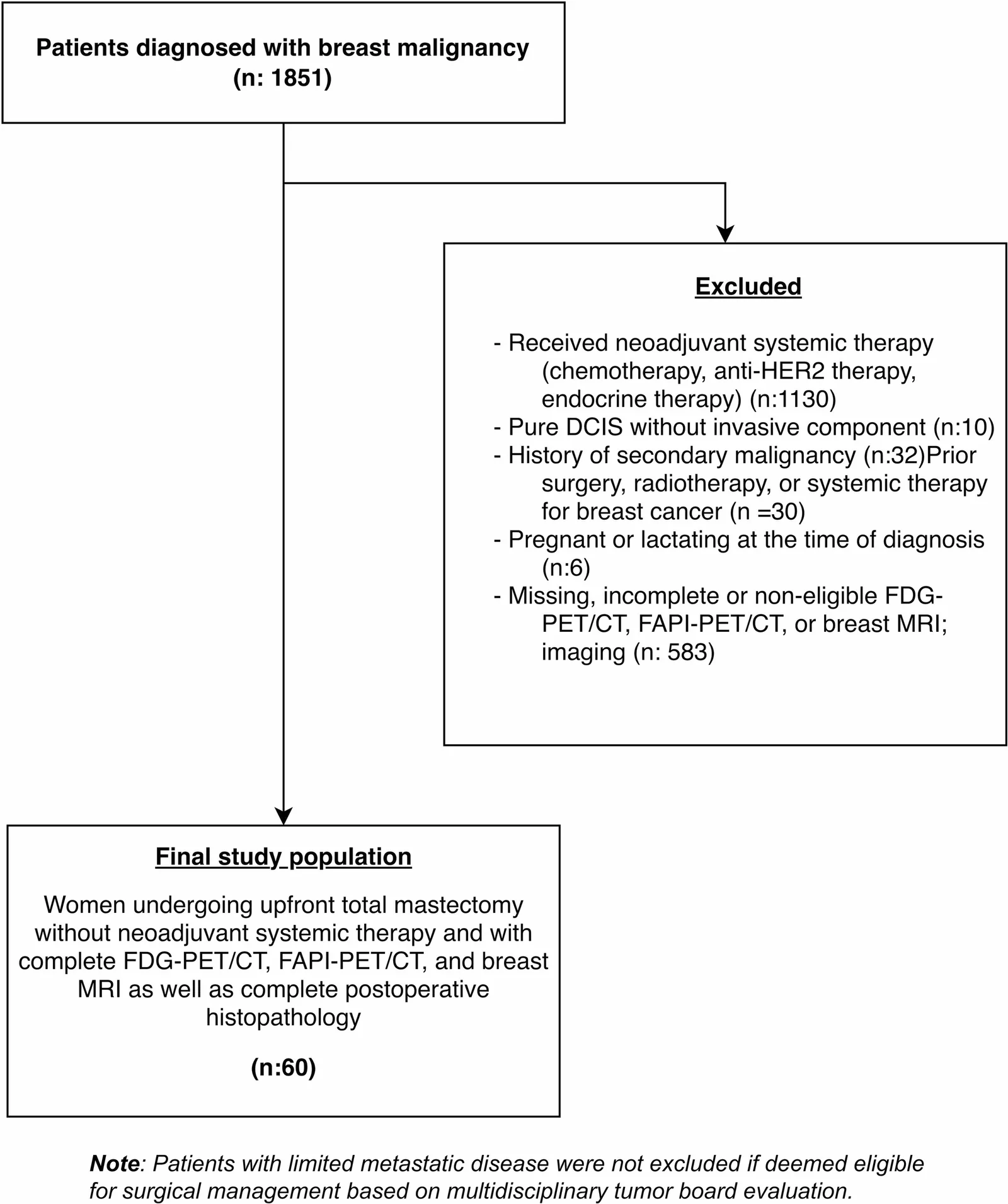
Flowchart of patient selection, exclusion criteria, and final study cohort

Patients were excluded if they had received any form of neoadjuvant systemic therapy, including chemotherapy, endocrine therapy, or targeted therapy, had prior breast cancer–related surgery, radiotherapy, or systemic therapy; had a history of secondary malignancy; were diagnosed with ductal carcinoma in situ (DCIS) only; underwent breast-conserving surgery (BCS); had known contraindications to gadolinium or ⁶⁸Ga-FAPI agents; lacked ¹⁸F-FDGPET/CT, ⁶⁸Ga-FAPIPET/CT, or breast MRI examinations; had unavailable imaging or postoperative pathology records in the institutional PACS archive; or were younger than 18 years (Fig. 1).

The median interval between FDG, FAPI, and MRI examinations was 7 days (Interquartile Range (IQR), 2–12), and the median time from the first imaging study to surgery was 16 days (IQR, 8–30). The study was approved by the local ethics committee (Ethics Committee No: 2024/377, dated 30 April 2025).

### Imaging Protocols and Image Evaluation

#### ⁶⁸Ga-FAPI-04 (FAPI) PET/CT

⁶⁸Ga-FAPI-04 was synthesized on-site using the Modular-Lab Easy automated platform (Eckert & Ziegler, Berlin, Germany), following standardized and validated procedures [26]. Radiochemical purity exceeded 98% in all syntheses. After quality control, an intravenous dose of 2–3 MBq/kg of ⁶⁸Ga-FAPI-04 was administered. Whole-body PET/CT imaging was performed 30–60 min after tracer injection, covering the skull base to the mid-thigh.

#### ¹⁸F-FDG PET/CT

¹⁸F-FDG was obtained from an external GMP-certified cyclotron facility and underwent routine quality control upon arrival. All patients fasted for 4–6 h, and only those with blood glucose < 150 mg/dL were scanned. An intravenous dose of 3.5–5.5 MBq/kg of ¹⁸F-FDG was administered. PET/CT acquisitions were performed on a Discovery IQ system (GE Healthcare, Milwaukee, WI, USA) using standard clinical parameters.

#### Common PET/CT Acquisition and Evaluation (FAPI and FDG)

For both ⁶⁸Ga-FAPI and ¹⁸F-FDG PET/CT studies, images were reconstructed using hybrid algorithms and reviewed in axial, coronal, and sagittal planes. Pathologic uptake was defined as focal radiotracer accumulation greater than background activity and interpreted within clinical context.

For each patient, the following variables were recorded: SUVmax and longest axial diameter of the primary tumor; presence of pathologic axillary lymph nodes; additional ipsilateral or contralateral intramammary tumor foci; and uptake in lymph nodes outside the MRI field of view, including level-3 axillary nodes and internal mammary nodes, as well as evidence of distant metastatic disease.

All PET/CT examinations were independently interpreted by two blinded nuclear medicine physicians, with discrepancies resolved by consensus.

Distant metastatic lesions were defined based on concordant findings on multimodal imaging (including ultrasonography, CT and/or MRI) and multidisciplinary clinical assessment when histopathological confirmation was not available.

#### Breast Magnetic Resonance Imaging (MRI)

All breast MRI examinations were performed on a 3.0-T scanner (Philips Healthcare, The Netherlands) using a dedicated bilateral breast coil. The protocol included non–fat-suppressed and fat-suppressed T1-weighted sequences, fat-suppressed T2-weighted imaging, diffusion-weighted imaging (DWI), and dynamic contrast-enhanced (DCE) imaging. For DCE sequences, a gadolinium-based contrast agent (0.1 mmol/kg) was administered intravenously using an automated injector.

All MRI studies were interpreted according to the ACR BI-RADS MRI Lexicon (5th edition) [27]. Axillary lymph nodes were evaluated using the Node-RADS classification, with Node-RADS ≥ 3 considered suspicious for axillary metastasis [28]. Because the standard breast MRI field of view primarily includes level 1 and level 2 axillary regions, and internal mammary nodes, only these nodal levels were assessed on MRI. Extra-axillary and level-3 axillary nodes were evaluated exclusively on PET/CT.

All MRI examinations were independently interpreted in a blinded manner by two breast radiologists (one of whom is an author of this study, with over 20 years of experience, and the other an experienced breast radiologist not involved in the authorship), with discrepancies resolved by consensus.

#### Reference Standard for Axillary Lymph Node Status

Axillary surgical management was performed in accordance with current NCCN and ESMO guidelines. In patients with suspicious axillary lymph nodes on preoperative imaging (*n* = 31), ultrasound-guided percutaneous core biopsy was performed prior to surgery. In patients with biopsy-confirmed metastatic involvement and appropriate clinical indication, axillary lymph node dissection (ALND) was planned following multidisciplinary tumor board evaluation (*n* = 25). In selected cases with limited nodal disease identified on multimodal imaging (¹⁸F-FDG PET/CT, ⁶⁸Ga-FAPI PET/CT, ultrasound, and MRI), targeted axillary dissection (TAD) was performed after clip placement (*n* = 6).

In patients without suspicious axillary findings on preoperative imaging (*n* = 29), sentinel lymph node biopsy (SLNB) was performed as the initial surgical approach. In our institution, intraoperative frozen section analysis is routinely performed during SLNB; in cases with extensive nodal involvement (*n* = 6), conversion to ALND was performed intraoperatively, while the remaining patients (*n* = 23) underwent SLNB alone.

All patients in the study cohort underwent upfront surgery without neoadjuvant therapy, and axillary lymph node status was determined by postoperative histopathological examination in all cases. Therefore, histopathology served as the reference standard for axillary staging, and no patient was evaluated based on imaging or clinical follow-up alone.

#### Statistical Analysis

All statistical analyses were performed using IBM SPSS Statistics (version 31.0; IBM Corp., Armonk, NY, USA) and R software (version 4.4.2; R Foundation for Statistical Computing, Vienna, Austria). Continuous variables were assessed for normality using the Shapiro–Wilk test and are reported as mean ± standard deviation (SD) or median with interquartile range (IQR), as appropriate. Categorical variables are presented as absolute frequencies and percentages.

Comparisons of tumor size measurements obtained by ¹⁸F-FDG PET/CT, ⁶⁸Ga-FAPI PET/CT, and breast MRI were conducted using the Friedman test for paired non-parametric samples. When the overall test was significant, pairwise Wilcoxon signed-rank tests with Bonferroni correction were applied for post-hoc comparisons. Associations between imaging-derived tumor size measurements and histopathological tumor size were evaluated using Spearman’s rank correlation coefficient (ρ).

For the assessment of tumor focality (unifocal, multifocal, multicentric), pathology was used as the reference standard. Differences in the distribution of focality classifications across imaging modalities were analyzed using Cochran’s Q test for paired categorical data, followed by pairwise McNemar tests (exact test when applicable) for post-hoc comparisons. Agreement between each imaging modality and histopathology was quantified using Cohen’s κ coefficient, along with the percentage of overall agreement. κ values were interpreted according to the Landis and Koch criteria (≤ 0.20 = slight, 0.21–0.40 = fair, 0.41–0.60 = moderate, 0.61–0.80 = substantial, ≥ 0.81 = almost perfect agreement).

For axillary lymph-node status, histopathology served as the reference standard. Diagnostic performance metrics, including sensitivity, specificity, positive predictive value (PPV), negative predictive value (NPV), and overall accuracy, were calculated for each imaging modality based on true-positive, true-negative, false-positive, and false-negative classifications.

While the Friedman test was used to compare tumor size measurements across imaging modalities, correlation analyses were performed separately to assess the relationship between imaging-based and pathologic tumor size measurements. For tumor focality, agreement statistics were preferred over diagnostic accuracy metrics due to the multicategory nature of the outcome.

Interobserver agreement was evaluated using intraclass correlation coefficients (ICC) for continuous variables (two-way random-effects model, absolute agreement) and Cohen’s κ statistics for categorical parameters, with corresponding 95% confidence intervals. A two-sided p value < 0.05 was considered statistically significant.

## Results

### Patient and Tumor Characteristics

A total of 60 patients (mean age, 53.7 ± 11.54 years) were included in the study. Tumors were almost evenly distributed between the left (51.7%) and right (48.3%) breasts, with the upper-outer quadrant being the most common tumor location (40.0%), followed by the lower-outer (26.7%) and upper-inner quadrants (20.0%) (Table 1).

**Table 1:** Baseline clinicopathologic characteristics of the study cohort

Variable	Value / Distribution
Age (years), mean ± SD	53.7 ± 11.54
Tumor laterality	Left: 31 (51.7%)Right: 29 (48.3%)
Tumor quadrant	Upper-outer (UOQ): 24 (40.0%) Lower-outer (LOQ): 16 (26.7%) Upper-inner (UIQ): 12 (20.0%) Lower-inner (LIQ): 5 (8.3%) Central / Retroareolar: 3 (5.0%)
Histopathological type	IDC: 49 (81.7%) ILC: 7 (11.7%) Mixed: 4 (6.7%)
Histologic grade	Grade 1: 6 (10.0%) Grade 2: 35 (58.3%) Grade 3: 19 (31.7%)
Lymphovascular invasion	Absent: 53 (88.3%) Present: 7 (11.7%)
Multifocality/multicentricity (based on post-operative pathology)	Unifocal: 32 (53.3%)Multifocal (MF): 17 (28.3%) Multicentric (MC): 11 (18.3%)
Accompanying DCIS	Absent: 33 (55.0%) Present: 27 (45.0%)
Estrogen receptor (ER)	Positive: 57 (95.0%) Negative: 3 (5.0%)
Progesterone receptor (PR)	Positive: 53 (88.3%) Negative: 7 (11.7%)
HER2 receptor	Positive: 5 (8.3%) Negative: 55 (91.7%)
Ki-67 (%), median (IQR)	15 (10–24)
Molecular subtype	Luminal A: 32 (53.3%) Luminal B: 24 (40.0%) HER2-enriched: 2 (3.3%) Triple-negative: 2 (3.3%)
Pathologic tumor size (mm), median (IQR)	23 (15–34)
Axillary lymph node status based on post-operative pathology	Positive: 16 (26.7%) Negative / Reactive: 44 (73.3%)
Axillary lymph node dissection (ALND)	Yes: 31 (51.7%) No: 29 (48.3%) (6 targeted axillary dissection [TAD], 23 SLNB alone)

Among the 60 patients, axillary lymph node dissection (ALND) was performed in 31 patients, including 25 patients with biopsy-proven nodal metastasis and 6 patients who underwent intraoperative conversion to ALND following positive sentinel lymph node biopsy (SLNB) based on intraoperative frozen section assessment. SLNB alone was performed in 23 patients. Targeted axillary dissection (TAD) was performed in 6 patients with limited nodal disease following preoperative clip placement of biopsy-proven metastatic lymph nodes.

Histopathological evaluation revealed invasive ductal carcinoma (IDC) in 81.7% of cases, ILC in 11.7%, and mixed histology in 6.7%. Most tumors were of intermediate or high histologic grade (Grade 1, 10%; Grade 2, 58.3%; Grade 3, 31.7%), and lymphovascular invasion was present in 11.7% of patients. Multifocal disease was identified in 28.3% and multicentric disease in 18.3% of patients, with an accompanying DCIS component observed in 45.0%.

Hormone receptor positivity was high, with estrogen receptor (ER) positivity in 95.0% and progesterone receptor (PR) positivity in 88.3%, whereas HER2 positivity was observed in 8.3%. Luminal subtypes predominated (Luminal A, 53.3%; Luminal B, 40.0%), while HER2-enriched and triple-negative subtypes were rare (3.3% each). The median Ki-67 index was 15% (IQR, 10–24). The median pathologic tumor size was 23 mm (IQR, 15–34). Postoperative histopathology confirmed axillary lymph node metastasis in 26.7% of patients (Table 1).

### Imaging Findings by Modality

Descriptive imaging findings for ¹⁸F-FDG PET/CT, ⁶⁸Ga-FAPI PET/CT, and breast MRI are summarized in Table 2. The median tumor size measured by imaging was 18.5 mm (IQR, 0–30) on FDG-PET, 22 mm (IQR, 13–35.75) on FAPI-PET, and 25 mm (IQR, 12.75–39) on breast MRI.

Unifocal disease predominated across all modalities, most frequently on FDG-PET (81.7%), followed by FAPI-PET (61.7%) and breast MRI (55.0%). Multifocal and multicentric disease were more frequently identified by FAPI-PET (23.3% and 15.0%, respectively) and breast MRI (26.7% and 18.3%) compared with FDG-PET (10.0% and 8.3%) (Table 2).

**Table 2:** Descriptive imaging findings by modality

Variable	FDG-PET	FAPI-PET	Breast MRI
Tumor size (mm), median (IQR)	18.5 ( 0–30 )	22 ( 13–35.75 )	25 ( 12.75–39 )
Additional focus			
Unifocal (UF)	49 (81.7%)	37 (61.7%)	33 (55.0%)
Multifocal (MF)	6 (10.0%)	14 (23.3%)	16 (26.7%)
Multicentric (MC)	5 (8.3%)	9 (15.0%)	11 (18.3%)
SUVmax, median (IQR)	3.25 (0–5.5 )	11.65 ( 7.38–16.33 )	—
Axillary status			
Positive	10 (16.7%)	13 (21.7%)	26 (43.3%)
Negative	50 (83.3%)	47 (78.3%)	34 (56.7%)
Morphologically suspicious LAP without pathologic uptake			
Yes	14 (23.3%)	14 (23.3%)	—
No	46 (76.7%)	46 (76.7%)	—
Level 3 lymph node involvement	2 (3.3%)	3 (5.0%)	—
Internal mammary LAP	—	—	—
Distant metastasis	0 (0%)	4 (6.7%)	—
FAPI additional findings vs. FDG	—	7 (11.7%) additional axillary LAPs	—
FAPI additional foci in same/contralateral breast	—	29 (48.3%)	—

Distant metastatic findings detected by ⁶⁸Ga-FAPI PET/CT were observed in four patients and included: a subcapsular hepatic lesion (histopathologically confirmed) with concomitant left rib involvement, an isolated iliac bone lesion, isolated calvarial involvement, and isolated lumbar vertebral involvement. All patients with additional metastatic findings had luminal A subtype breast cancer. Bone lesions were interpreted as metastatic based on concordant findings on multimodal imaging and multidisciplinary tumor board evaluation. These cases were considered within an oligometastatic disease setting. Following patient counseling, treatment strategies were individualized, and surgical management was pursued within a multidisciplinary decision-making framework

The median SUVmax of the primary tumor was higher on FAPI-PET (11.65; IQR, 7.38–16.33) than on FDG-PET (3.25; IQR, 0–5.5) (Table 2). Visually appreciable FDG uptake at the primary tumor site was absent in 18 of 60 patients (30.0%), although invasive disease was confirmed histopathologically.

Regarding axillary assessment, metastatic lymph node positivity was observed in 16.7% of FDG-PET and 21.7% of FAPI-PET examinations, whereas breast MRI demonstrated axillary positivity in 43.3% of cases. Morphologically suspicious but metabolically inactive lymph nodes were observed in 23.3% (n:14) of both FDG-PET and FAPI-PET studies. Level-3 axillary lymph node involvement was identified in 3.3% of FDG-PET and 5.0% of FAPI-PET examinations. Internal mammary chain involvement was not detected on any modality. Distant metastases were detected in four patients (6.7%) on FAPI-PET, whereas the corresponding lesions were either not visualized or showed only subtle uptake on FDG-PET. (Table 2). Among the four patients in whom FAPI-PET/CT identified additional distant metastatic findings, the distribution was as follows: in one patient, a subcapsular hepatic lesion (histopathologically confirmed) with concomitant involvement of the left rib; in another patient, an isolated iliac bone metastasis; in a third patient, isolated calvarial bone involvement; and in the remaining patient, isolated lumbar vertebral involvement. Following multidisciplinary tumor board evaluation and patient counseling, treatment decisions were individualized, and surgical management was considered in selected cases.

### Correlation and Agreement with Pathologic Tumor Size

**Fig. 2:**
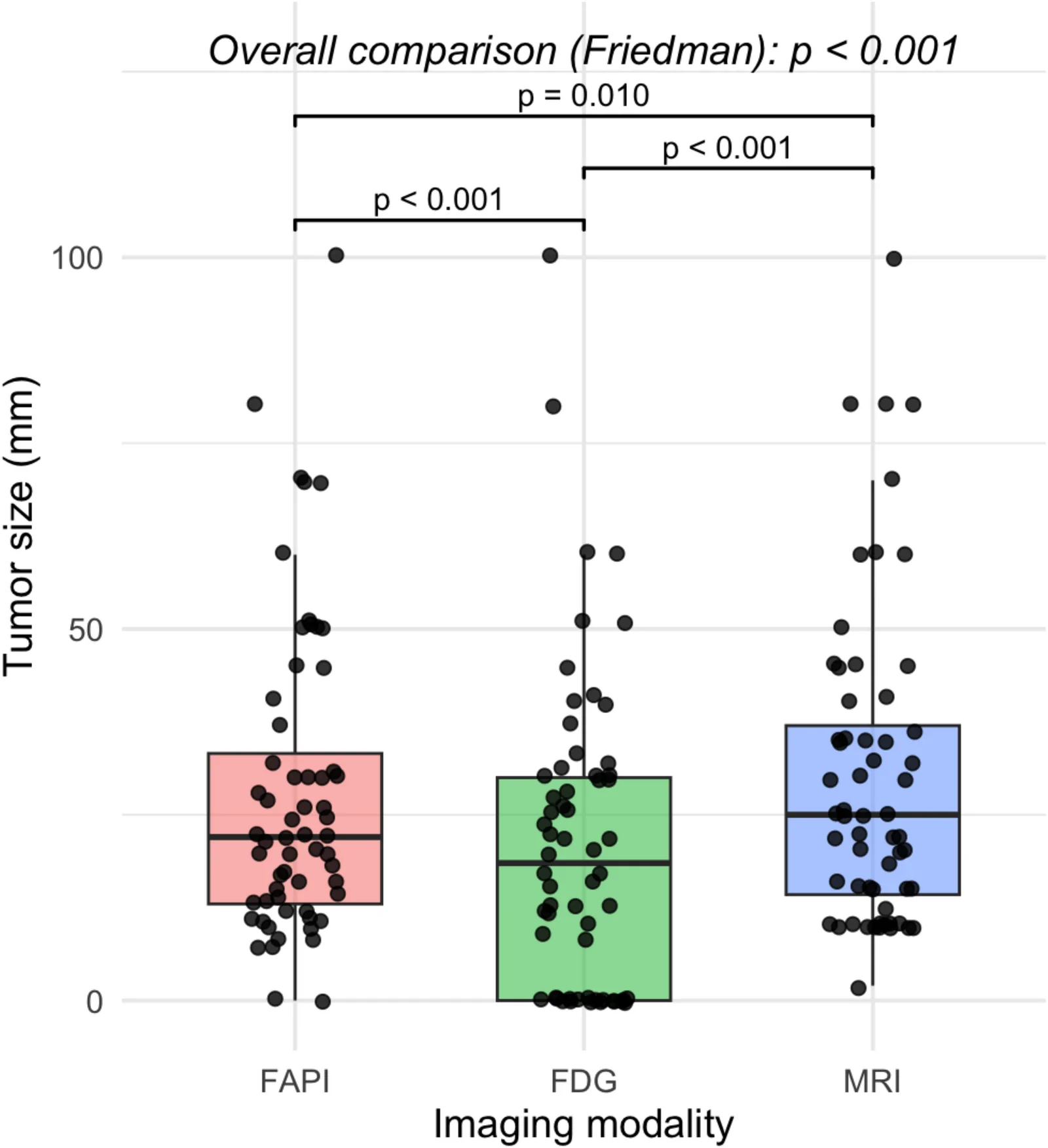
Paired comparison of tumor size measurements obtained by ¹⁸F-FDGPET/CT, ⁶⁸Ga-FAPIPET/CT, and breast MRI. Boxplots show median and interquartile range, with individual patient measurements overlaid. Overall and pairwise statistical comparisons were performed using Friedman and Wilcoxon signed-rank tests, respectively

Tumor size measurements differed significantly among FDG-PET, FAPI-PET, and breast MRI (Friedman test, *p* < 0.001), as illustrated in Fig. 2. Post-hoc Wilcoxon signed-rank analyses demonstrated that both FAPI-PET and breast MRI measured significantly larger tumor sizes than FDG-PET (*p* < 0.001 and *p* = 0.010, respectively). Breast MRI also yielded significantly larger measurements than FAPI-PET (*p* < 0.001).

Correlation analysis revealed strong positive associations between imaging-derived and pathologic tumor size for all modalities (Table 3). Breast MRI showed the strongest correlation (Spearman’s ρ = 0.866, *p* < 0.001), followed closely by FAPI-PET (ρ = 0.844, *p* < 0.001), whereas FDG-PET demonstrated a weaker but still significant correlation (ρ = 0.780, *p* < 0.001).

**Table 3:** Correlation between imaging-derived and histopathological tumor size

Imaging modality	Spearman *r*	*p*-value
FDG-PET	0.780	< 0.001
FAPI-PET	0.844	< 0.001
Breast MRI	0.866	< 0.001

### Comparative Analysis of Lesion Detection (Primary, Multifocal, and Multicentric Lesions)

Histopathological examination confirmed multifocal or multicentric disease in 46.7% of patients. Differences in the detection of multifocal or multicentric disease among imaging modalities were statistically significant (Cochran’s Q = 20.8, df = 2, *p* < 0.001) (Table 4).

**Table 4:** Detection performance and agreement for multifocal/multicentric disease

Comparison between modalities			
Analysis			Result
Cochran’s Q test			Q = 20.8, df = 2, *p* < 0.001
FDG-PET vs. FAPI-PET			*p* < 0.001
FDG-PET vs. Breast MRI			*p* < 0.001
FAPI-PET vs. Breast MRI			*p* = 0.289
Consistency with pathology			
Modality	MF/MC detected n (%)	Agreement with pathology (%)	Cohen’s κ (p)
FDG-PET	11 (18.3%)	71.7%	0.408 (< 0.001)
FAPI-PET	23 (38.3%)	91.7%	0.831 (< 0.001)
Breast MRI	27 (45.0%)	95.0%	0.899 (< 0.001)

Binary classification was used for focality assessment (0 = unifocal, 1 = multifocal or multicentric). A Bonferroni-adjusted significance threshold of *p* < 0.016 was applied for post-hoc McNemar tests

FDG-PET identified multifocal or multicentric disease in 11 patients (18.3%), yielding an overall agreement of 71.7% with histopathology and moderate agreement (κ = 0.408, *p* < 0.001). FAPI-PET detected multifocal or multicentric disease in 23 patients (38.3%), with an agreement rate of 91.7% and almost perfect agreement with pathology (κ = 0.831, *p* < 0.001). Breast MRI demonstrated the highest detection rate, identifying multifocal or multicentric disease in 27 patients (45.0%), corresponding to an agreement of 95.0% and almost perfect concordance (κ = 0.899, *p* < 0.001) (Table 4).

**Fig. 3:**
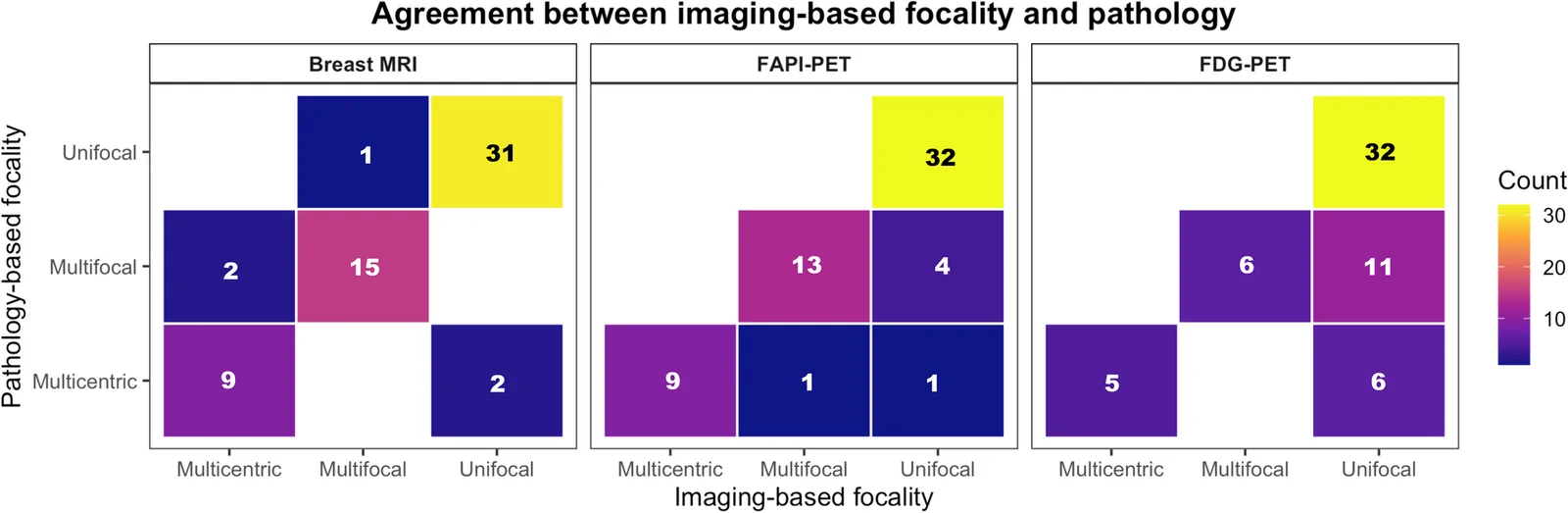
Heatmap representation of agreement between imaging-based and pathology-based focality classifications across FDG-PET, FAPI-PET, and breast MRI

Pairwise comparisons showed that both FAPI-PET and breast MRI detected additional intramammary foci significantly more frequently than FDG-PET (both *p* < 0.001), whereas no significant difference was observed between FAPI-PET and breast MRI (*p* = 0.289). The distribution of focality classifications across imaging modalities relative to pathologic findings is illustrated in Fig. 3.

### Axillary Lymph Node Status and Diagnostic Performance

**Fig. 4:**
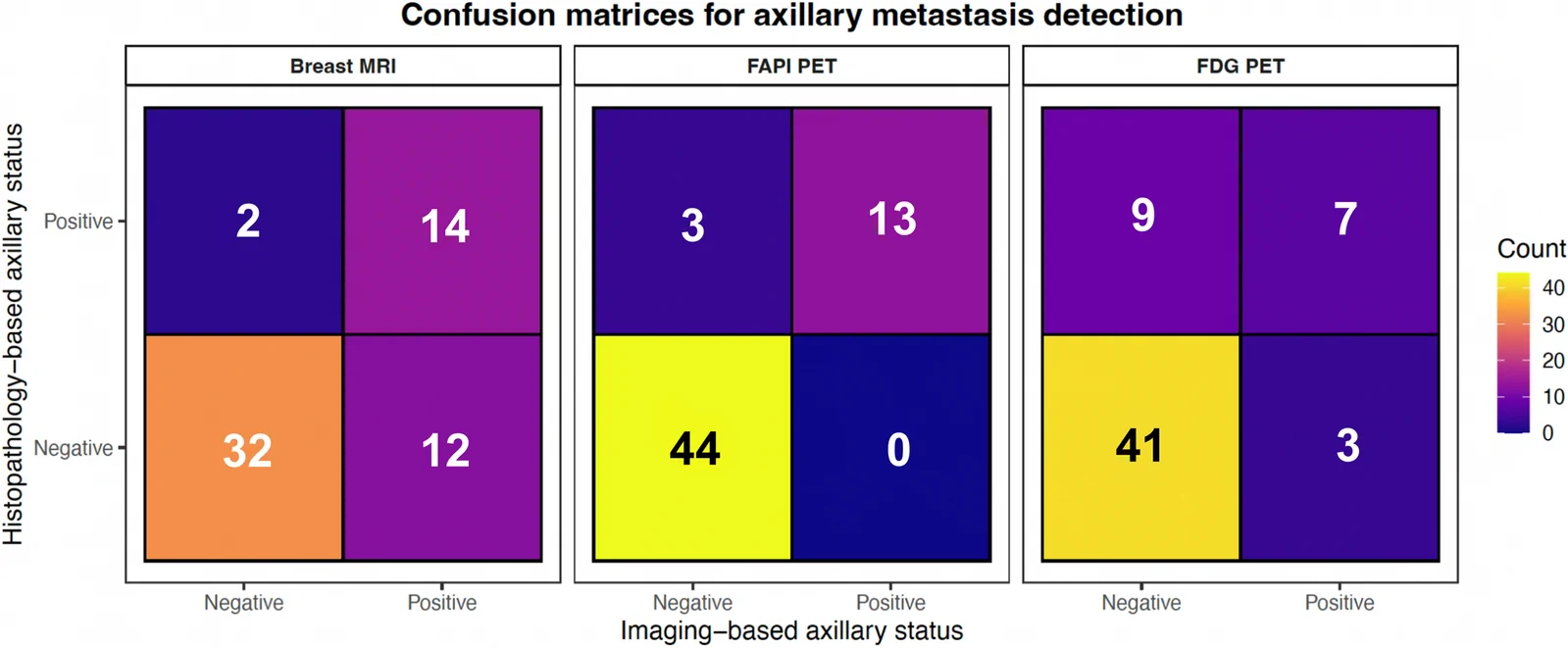
Confusion matrices for axillary metastasis detection by imaging modality

**Fig. 5:**
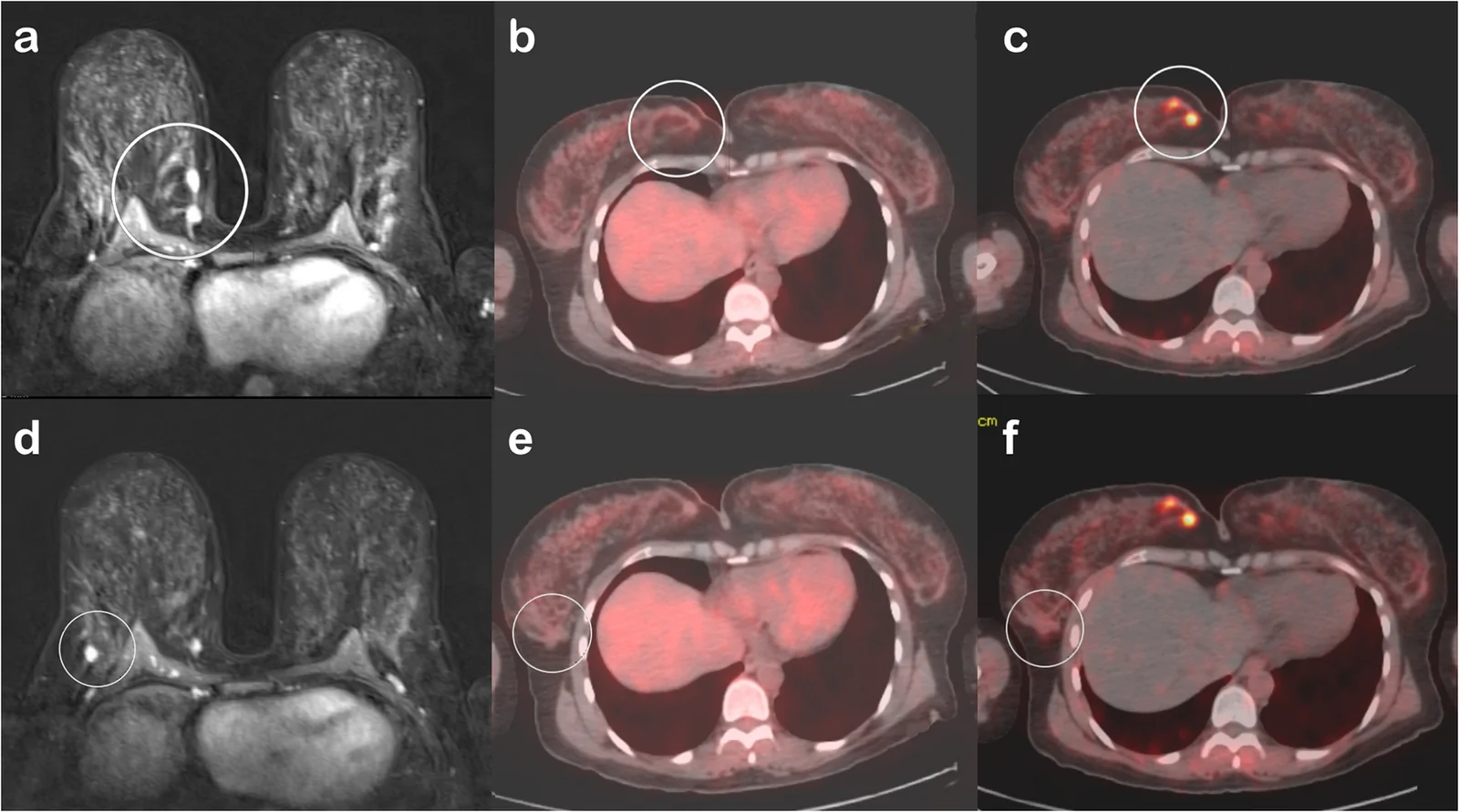
A 42-year-old woman with IDC of the right breast. Breast MRI demonstrates two adjacent enhancing lesions in the lower-inner quadrant measuring 10 mm and 8 mm, corresponding to the primary tumor and an additional focus (multifocal) (**a**). On ¹⁸F-FDG PET/CT, no appreciable radiotracer uptake is observed in either lesion (**b**). In contrast, ⁶⁸Ga-FAPI PET/CT shows intense pathologic radiotracer uptake in both lesions, each measuring approximately 10 mm, with a SUVmax of 10 (**c**). Additionally, breast MRI reveals an 8 mm enhancing lesion in the lower-outer quadrant, representing a multicentric focus (**d**). This multicentric lesion shows no abnormal uptake on either ¹⁸F-FDG PET/CT (**e**) or ⁶⁸Ga-FAPI PET/CT (**f**). The patient underwent skin-sparing mastectomy without neoadjuvant chemotherapy. Postoperative histopathology revealed IDC, 10 mm, ER 85%, PR 95%, HER2 negative, Ki-67 10%, grade 2. The multicentric lesion in the lower-outer quadrant demonstrated an identical biological profile. Corresponding lesions are indicated by circles across modalities

**Fig. 6:**
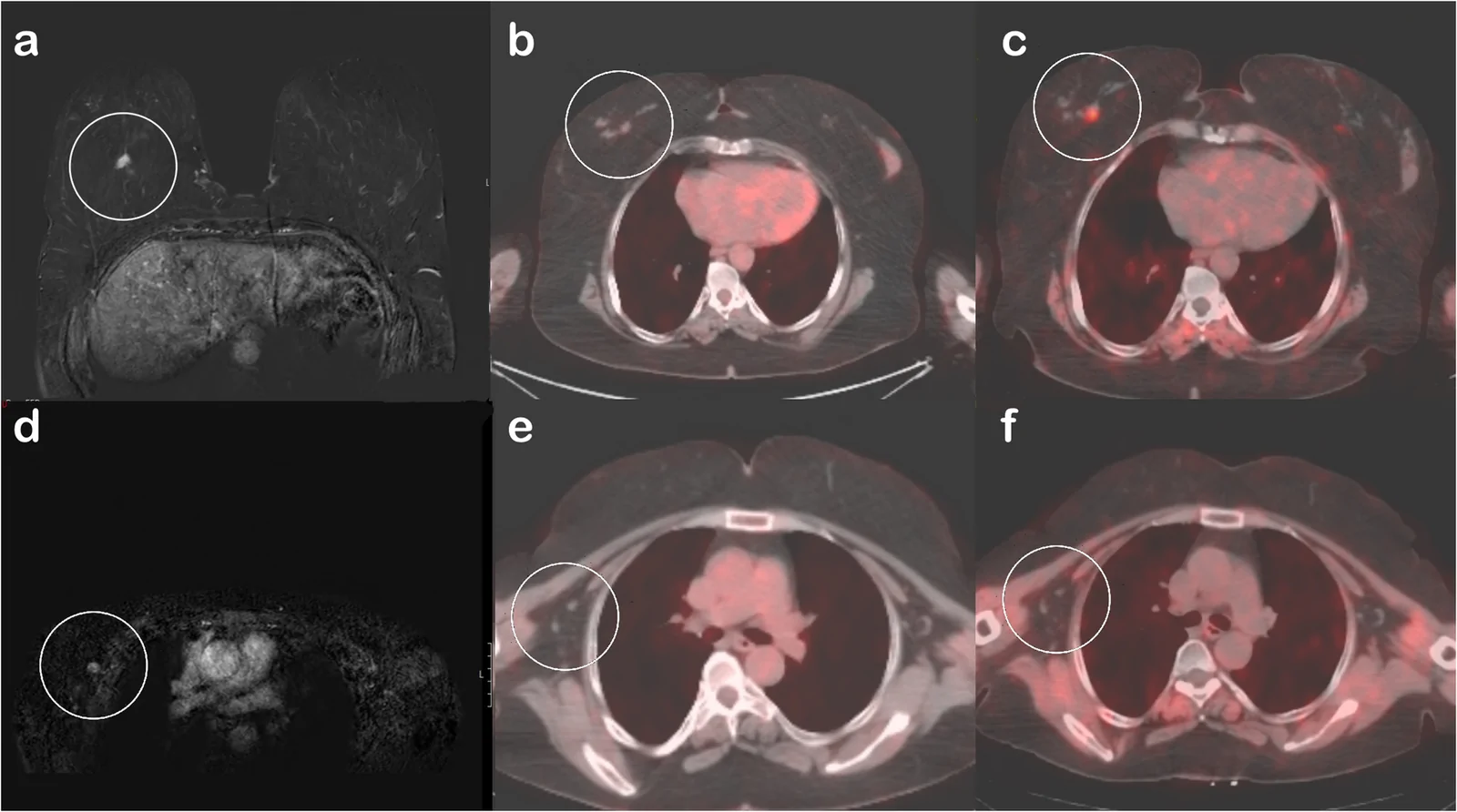
A 60-year-old postmenopausal woman with IDC of the right breast. Breast MRI demonstrates a 14 × 7 mm enhancing lesion at the 6 o’clock position consistent with the primary malignancy (**a**). No appreciable uptake is observed at this site on ¹⁸F-FDG PET/CT (**b**), whereas ⁶⁸Ga-FAPI PET/CT shows pathologic radiotracer accumulation measuring approximately 12 mm (SUVmax: 5.8), concordant with MRI (**c**). Preoperative MRI also identified a small, spherical, suspicious lymph node in the right axilla (**d**); however, no abnormal uptake was detected in this node on either ¹⁸F-FDG PET/CT (**e**) or ⁶⁸Ga-FAPI PET/CT (**f**). Ultrasound-guided fine-needle aspiration performed due to the MRI finding was negative for metastatic involvement. The patient underwent total mastectomy (patient preference) without neoadjuvant therapy. Postoperative histopathology revealed IDC measuring 15 mm, ER 90%, PR 90%, HER2 negative, Ki-67 5%, grade 2. Sentinel lymph node biopsy showed no metastatic axillary involvement. Corresponding lesions are indicated by circles across modalities

**Fig. 7:**
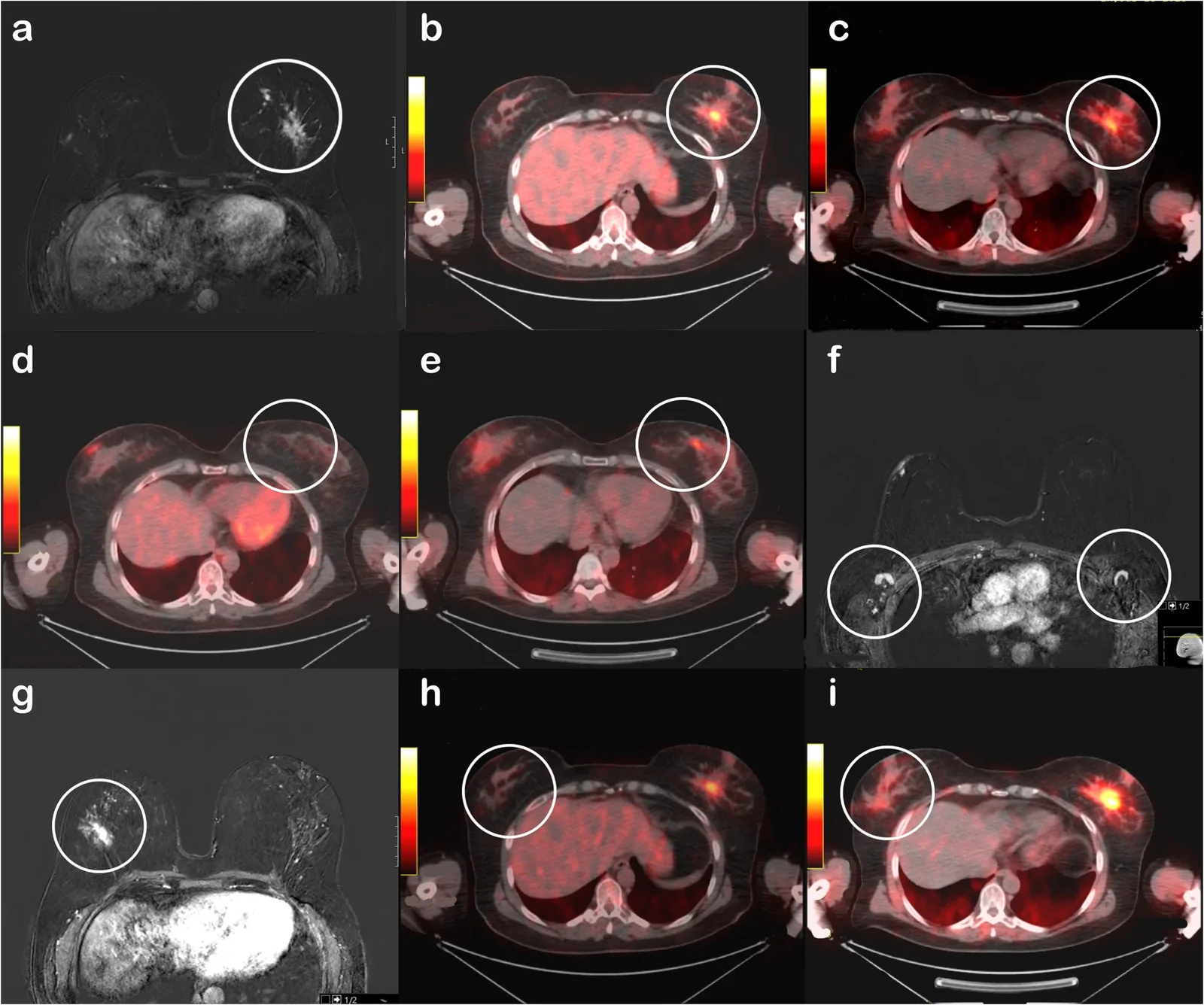
A 50-year-old premenopausal patient with biopsy-proven ILC of the left breast. Breast MRI shows a 35 × 20 mm primary tumor in the outer central left breast, accompanied by several small adjacent satellite foci medially (**a**). On ¹⁸F-FDG PET/CT, the primary lesion demonstrates a markedly underestimated metabolic diameter of approximately 10 mm with an SUVmax of 6 (**b**). In contrast, ⁶⁸Ga-FAPI PET/CT depicts a larger extent of involvement measuring approximately 15 mm with intense radiotracer uptake (SUVmax: 18) (**c**). While ¹⁸F-FDG PET/CT fails to demonstrate pathologic uptake in any of the additional foci (**d**), ⁶⁸Ga-FAPI PET/CT reveals increased peritumoral activity and focal uptake corresponding to one of the satellite lesions (**e**). MRI, however, identifies a greater number of multifocal lesions than either PET modality (**a**). Axillary evaluation on MRI demonstrated several bilateral lymph nodes with mildly thickened cortices but preserved fatty hila; neither ¹⁸F-FDG PET/CT nor ⁶⁸Ga-FAPI PET/CT showed pathologic uptake in these nodes in either axilla (**f**). Additionally, MRI detected a 30 × 10 mm segment of non-mass enhancement in the right breast (**g**). This region showed no appreciable uptake on FDG (**h**), whereas ⁶⁸Ga-FAPI PET/CT demonstrated only minimal parenchymal activity (**i**). The patient underwent bilateral skin-sparing mastectomy without neoadjuvant therapy. Postoperative pathology revealed bilateral ILC (left: 40 mm; right: 35 mm), ER 95%, PR 95%, HER2 negative, Ki-67 5%, grade 1. No axillary metastatic involvement was identified in the sentinel lymph node biopsy

**Fig. 8:**
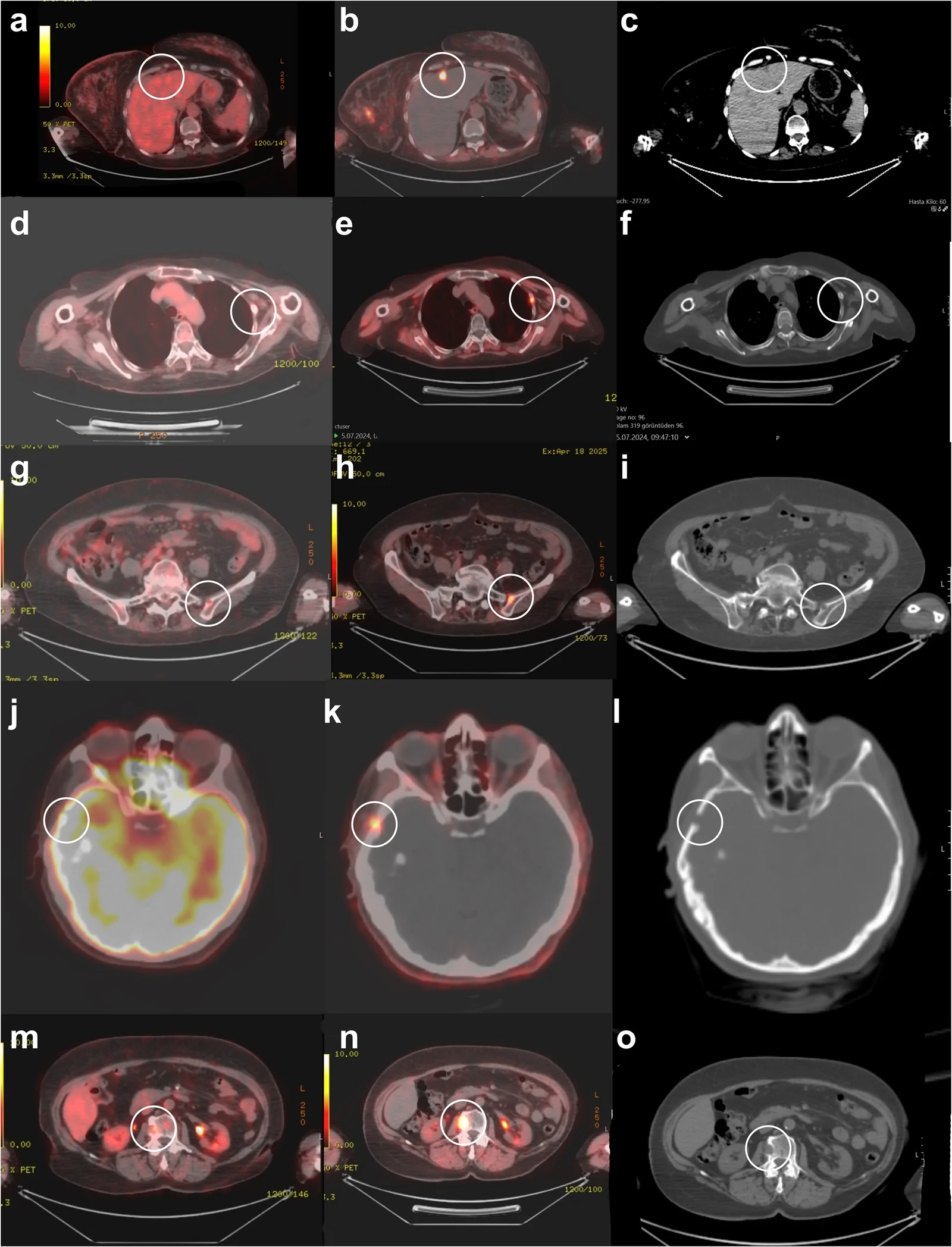
Representative cases of additional distant metastatic lesions detected by ⁶⁸Ga-FAPI PET/CT but not clearly identified on ¹⁸F-FDG PET/CT. In a 67-year-old woman, no definite pathologic uptake was observed in the liver on FDG PET/CT (**a**), whereas ⁶⁸Ga-FAPI PET/CT demonstrated a subcapsular nodular lesion measuring approximately 8 mm with increased uptake (SUVmax: 14.7) (**b**). Corresponding native CT confirmed a subcapsular, ill-defined hypodense lesion (**c**). Subsequent ultrasound-guided core needle biopsy confirmed metastatic involvement. In the same patient, a lesion in the left lateral rib showed no FDG uptake (**d**), while ⁶⁸Ga-FAPI PET/CT revealed increased uptake (SUVmax: 9.6) (**e**). Native CT demonstrated an expansile, soft-tissue-density lesion causing cortical disruption (**f**). In a 70-year-old woman, a lesion in the left iliac bone demonstrated subtle uptake on ¹⁸F-FDG PET/CT (SUVmax: 5) (**g**), whereas ⁶⁸Ga-FAPI PET/CT showed markedly increased uptake (SUVmax: 10.7) (h). Native CT revealed a small cortical-destructive lesion consistent with osseous metastasis (i). In a 58-year-old woman, no definite FDG uptake was observed in the calvarium (**j**), whereas ⁶⁸Ga-FAPI PET/CT demonstrated focal increased uptake (SUVmax: 8.8) in the right temporal bone (**k**). Corresponding native CT showed a lytic bone lesion at the same location (**l**). In a 62-year-old woman, a lesion in the right aspect of the L3 vertebral body showed subtle uptake on ¹⁸F-FDG PET/CT (SUVmax: 8) (**m**), while ⁶⁸Ga-FAPI PET/CT demonstrated markedly increased uptake (SUVmax: 15.7) (**n**). Native CT revealed a destructive lesion with mixed lytic-sclerotic changes and an associated soft-tissue component (**o**). All patients shown in this figure had luminal A subtype breast cancer. Among these lesions, only the hepatic lesion was histopathologically confirmed, whereas bone lesions were interpreted as metastatic based on concordant findings on multimodal imaging and multidisciplinary tumor board evaluation. Following patient counseling, treatment strategies were individualized, and these cases were managed within a multidisciplinary framework in the context of limited metastatic disease. Circles across modalities indicate corresponding lesions in all figures

Using postoperative histopathology as the reference standard, the diagnostic performance of breast MRI, FDG-PET, and FAPI-PET for detecting axillary lymph node metastasis is summarized in Table 5, with corresponding confusion matrices illustrated in Fig. 4. (Figures 5, 6, 7 and 8)

**Table 5:** Diagnostic Performance of Imaging Modalities for Axillary Metastasis (Pathology as Reference)

Modality	TP	TN	FP	FN	Sensitivity (%)	Specificity (%)	PPV (%)	NPV (%)	Accuracy (%)
Breast MRI	14	32	12	2	87.5	72.7	53.8	94.1	76.7
FDG PET	7	41	3	9	43.8	93.2	70.0	82.0	80.0
FAPI PET	13	44	0	3	81.2	100.0	100.0	93.6	95.0

Histopathology was used as the reference standard

*TP* true positive, *TN* true negative, *FP* false positive, *FN* false negative

*PPV* positive predictive value, *NPV* negative predictive value

Breast MRI demonstrated the highest sensitivity (87.5%), correctly identifying 14 of 16 node-positive patients, but showed lower specificity (72.7%) due to a higher number of false-positive findings. FDG-PET demonstrated high specificity (93.2%) and low sensitivity (43.8%), associated with an increased rate of false-negative results.

FAPI-PET demonstrated high sensitivity (81.2%) combined with perfect specificity (100.0%), with no false-positive axillary lymph nodes observed. This corresponded to the highest overall diagnostic accuracy (95.0%) (Table 5; Fig. 4).

### Interobserver Agreement

Interobserver agreement analyses were performed separately for breast MRI, ¹⁸F-FDG PET/CT, and ⁶⁸Ga-FAPI PET/CT for primary tumor measurements, detection of multifocal or multicentric foci, and assessment of axillary lymph node status. For breast MRI, agreement between the two radiologists ranged from substantial to almost perfect, with an intraclass correlation coefficient (ICC) of 0.88 (95% CI: 0.84–0.92) for tumor size measurements, substantial agreement for the detection of additional intramammary foci (κ = 0.76, *p* < 0.001), and substantial agreement for axillary lymph node evaluation (κ = 0.74, 95% CI: 0.66–0.82). For ¹⁸F-FDG PET/CT, interobserver agreement exceeded that of MRI, with excellent agreement for tumor SUVmax measurements (ICC = 0.92, 95% CI: 0.89–0.95), substantial agreement for detecting additional intramammary foci (κ = 0.78, *p* < 0.001), and almost perfect agreement for assessing axillary metastasis (κ = 0.81, 95% CI: 0.72–0.88). For ⁶⁸Ga-FAPI PET/CT, the highest interobserver agreement was observed, with an ICC of 0.95 (95% CI: 0.92–0.97) for tumor size measurements, almost perfect agreement for detecting additional intramammary foci (κ = 0.84, *p* < 0.001), and almost perfect agreement for axillary lymph node evaluation (κ = 0.87, 95% CI: 0.79–0.92). Additional assessments for higher-level lymph nodes and distant metastases showed κ values of 0.77 (FDG) and 0.86 (FAPI) for level 3 axillary nodes, 0.74 (FDG) and 0.85 (FAPI) for internal mammary lymph nodes, and 0.83 (FDG) and 0.91 (FAPI) for distant metastases.

## Discussion

In this study, ⁶⁸Ga-FAPI PET/CT, ¹⁸F-FDG PET/CT, and breast MRI were systematically compared across the key components of preoperative breast cancer staging, including primary tumor detection and tumor size assessment, detection of additional intramammary foci, and axillary nodal evaluation. By integrating quantitative size-correlation analyses, lesion-level detection performance, and nodal-staging accuracy, our findings provide a comprehensive overview of the relative contributions and limitations of each modality. The main results are discussed below under thematic domains.

### Primary Tumor Extent (T Stage)

In this study, the strongest correlation with pathologic tumor size was observed for breast MRI (ρ = 0.866) and ⁶⁸Ga-FAPI PET/CT (ρ = 0.844), indicating that both modalities provide highly accurate preoperative tumor size estimation. In contrast, ¹⁸F-FDG PET/CT demonstrated significantly weaker correlation (ρ = 0.780). Because FDG uptake is dependent on glucose metabolism and cellular proliferative activity, tumors with low metabolic rate, such as well-differentiated, low-proliferation, or certain histologic subtypes, may demonstrate minimal FDG uptake, thereby reducing size agreement with pathology [14, 16, 17]. In some cases, malignant lesions with low Ki-67 index, low mitotic activity, or lobular histology may even appear FDG-negative [14, 16, 17, 29]. Consistent with these biological constraints, visually appreciable FDG uptake at the primary tumor site was absent in 18 of 60 patients (30.0%), despite histopathological confirmation of invasive disease. This observation is in line with prior studies reporting low FDG avidity in ER-positive, low-proliferation, and lobular carcinomas, which together constituted the majority of our study population [14, 16, 17, 29].

Breast MRI, owing to its superior soft-tissue contrast and dynamic enhancement characteristics, demonstrated the highest detection rate for multifocal and multicentric disease (45.0%). However, FAPI-PET, through its unique mechanism based on stromal fibroblast activation, showed a comparable ability to identify additional foci (38.3%). This suggests that FAPI not only reflects metabolic activity but also captures tumor-associated stroma, enabling visualization of certain fibroblast-rich foci that may be occult on MRI [19, 20, 22, 30].

FDG-PET exhibited the lowest performance, detecting only 18.3% of multifocal/multicentric lesions. Although no subgroup analysis was performed for grade, Ki-67, or histologic subtype, prior studies have consistently reported reduced FDG sensitivity in low-grade or low-proliferation tumors and especially in ILC, where sensitivities may decline to 60% [16–18].

Overall, our findings indicate that FAPI-PET offers diagnostic performance superior to FDG and closely approaching MRI in terms of primary tumor extent and multiplicity. Given its rapid acquisition time and the absence of contraindications related to contrast agents or patient positioning, FAPI-PET may represent a practical alternative in patients unable to undergo MRI, for example, those with claustrophobia, severe obesity, contrast allergy, or limited MRI accessibility.

### Axillary Metastasis (N Stage)

In axillary staging, ⁶⁸Ga-FAPI PET/CT demonstrated high sensitivity combined with perfect specificity (sensitivity 81.2%, specificity 100.0%), outperforming ¹⁸F-FDG PET/CT (sensitivity 43.8%, specificity 93.2%) and achieving the highest overall diagnostic accuracy. Breast MRI showed the highest sensitivity (87.5%); however, its specificity was lower (72.7%). This finding is consistent with previous reports indicating that purely morphologic assessment, in the absence of functional imaging parameters, may lead to higher false-positive rates in axillary nodal evaluation [31, 32].

Although the limitations of ¹⁸F-FDG PET/CT in locoregional breast cancer assessment are well established, its inclusion enabled a direct head-to-head comparison with ⁶⁸Ga-FAPI PET/CT and breast MRI within the same post-operative histopathology-referenced cohort. Importantly, the clinical value of FAPI in axillary staging was primarily reflected by its ability to accurately predict histopathologically confirmed nodal status (N-positive vs. N-negative), rather than merely detecting a greater number of lymph nodes on imaging. Accordingly, axillary evaluation in this study primarily focused on accurate classification of nodal status rather than the number of detected lymph nodes.

Importantly, the predominance of luminal A/B subtypes (ER-positive, low Ki-67) in our cohort reflects a clinically relevant patient profile rather than incidental sampling bias. The stronger performance of FAPI-PET may be attributable to its ability to detect both high-proliferative and stroma-rich nodal metastases, owing to increased fibroblast activation within the tumor microenvironment. Prior evidence supports the superior contrast and broader uptake profile of FAPI compared with FDG in nodal metastases [23, 25].

Our study further showed that FAPI identified additional level-3 axillary nodes (5.0% vs. 3.3% with FDG) and detected distant metastasis in four patients, none of which were clearly identified on FDG-PET. These findings highlight the potential contribution of FAPI-PET in assessing metastatic spread beyond the conventional axillary basin. While FDG-PET provides high specificity in FDG-avid nodes, it may miss low-uptake or stromal-dominant nodal disease, which FAPI can capture.

### Clinical Implications and Practical Considerations

Taken together, the results suggest that ⁶⁸Ga-FAPI PET/CT may serve as a complementary modality to breast MRI and a valuable alternative to ¹⁸F-FDGPET/CT. FAPI appears particularly advantageous in tumors with substantial stromal components, ILC, and lesions with low FDG avidity.

FAPI-PET also provides practical workflow advantages, including short imaging time (30–60 min post-injection), absence of dietary preparation, and a characteristically high tumor-to-background ratio due to minimal physiological uptake in surrounding tissues, an aspect previously emphasized in the literature and reflected in our imaging observations [19–21].

### Interobserver Agreement

Interobserver analyses revealed modality-dependent variation in reproducibility. Breast MRI demonstrated substantial but comparatively lower agreement for tumor size, additional foci, and nodal assessment, likely influenced by variability in parenchymal enhancement, lesion morphology, and reader experience. ¹⁸F-FDG PET/CT showed higher consistency due to semi-quantitative SUV-based measurements and clearer lesion contrast. The highest overall agreement was observed with ⁶⁸Ga-FAPI PET/CT across all parameters, including primary tumor size, intramammary foci, and axillary or higher-level nodes, reflecting its high tumor-to-stroma contrast and minimal physiologic background uptake [19–21]. Collectively, these findings indicate that ⁶⁸Ga-FAPI PET/CT provides more reader-independent and consistent assessments than MRI or FDG, with meaningful implications for surgical planning and evaluation of multifocal or complex nodal patterns.

Although our study was not powered for subtype-specific analyses, existing literature demonstrates that histologic architecture can significantly influence molecular imaging performance. ILC, characterized by low cellular density, diffuse stromal infiltration, and frequent non-mass enhancement, often shows reduced FDG avidity and poses challenges for margin delineation on MRI [33–35]. Conversely, mucinous carcinomas, defined by abundant extracellular mucin and sparse cellularity, commonly exhibit reduced radiotracer uptake, resulting in lower conspicuity on both FDG- and FAPI-based imaging [36, 37]. These biologic considerations underscore the need for future studies specifically designed to evaluate stromal-dominant and mucin-rich tumor subtypes using ⁶⁸Ga-FAPI PET/CT and multiparametric MRI, to better identify patients who may derive the greatest benefit from FAPI-based staging.

### Limitations

This study has several limitations. First, its retrospective design and archive-based patient selection introduce the possibility of selection bias. Restricting the study population to patients treated with upfront total mastectomy may have indirectly contributed to a higher prevalence of multifocal or multicentric disease within the cohort. While this limits generalizability, this deliberate selection enabled comprehensive whole-breast histopathological evaluation, allowing a reliable one-to-one comparison between imaging findings and postoperative pathology. In this context, the increased prevalence of multifocal or multicentric tumors also provided an opportunity to more rigorously assess the performance of imaging modalities in detecting additional tumor foci under clinically challenging conditions. The relatively low incidence of grade 1 tumors in our cohort (10%) likely reflects this selection bias, as patients undergoing upfront total mastectomy are typically associated with more extensive or multifocal disease, resulting in a cohort predominantly composed of grade 2 and 3 tumors.

Second, the sample size was relatively limited (*n* = 60) and derived from a single center, which may limit external validity. The requirement for complete multimodal imaging (¹⁸F-FDG PET/CT, ⁶⁸Ga-FAPI PET/CT, and breast MRI) and the exclusion of patients receiving neoadjuvant therapy further reduced the eligible study population. Consequently, the distribution of molecular subtypes was imbalanced, with underrepresentation of HER2-positive and triple-negative breast cancers, which may have limited subtype-specific analyses.

Third, although postoperative histopathology served as the reference standard, a time interval between imaging and surgery may have led to minor changes in tumor size, potentially affecting imaging–pathology agreement. However, this effect is likely negligible, given the low prevalence of aggressive molecular subtypes in the cohort (< 10%) and the relatively short interval between imaging and surgery (median, 16 days; IQR, 8–30), reducing the likelihood of significant interval tumor progression.

Fourth, subgroup analyses based on biological tumor characteristics, such as molecular subtype, Ki-67 index, histologic type, and tumor grade, were not performed. Therefore, the biological basis of FDG–FAPI uptake differences could not be statistically evaluated. For breast MRI, axillary assessment relied solely on morphologic Node-RADS criteria, and functional imaging sequences (DWI, DCE) were not separately analyzed, which may have contributed to the lower specificity observed.

Fifth, because the cohort consisted exclusively of patients undergoing upfront surgery, representing a relatively low-risk population for distant metastasis, the comparison between ⁶⁸Ga-FAPI and ¹⁸F-FDG PET/CT for distant metastatic disease may not fully reflect their performance in advanced-stage settings. Additionally, although tumor-to-background ratios (TBR) were not included in this analysis, FAPI is known to provide substantially higher TBR compared with FDG due to minimal physiologic background uptake, which may enhance lesion conspicuity, particularly in low-metabolic tumors [38, 39]. Moreover, the MRI protocol did not include systematic evaluation of Level 3 axillary lymph nodes, limiting cross-modality comparison at these stations to PET-based techniques only. Finally, the study lacked prospective randomization, external validation, and long-term clinical follow-up.

Despite these limitations, several strengths should be highlighted. These include the use of postoperative histopathology as a robust reference standard, the head-to-head comparison of three advanced imaging modalities within the same patient cohort, and the provision of original clinical data on FAPI-PET.

## Conclusion

In this study, ⁶⁸Ga-FAPI PET/CT, ¹⁸F-FDG PET/CT, and breast MRI were compared using postoperative histopathology as the reference standard. ⁶⁸Ga-FAPI PET/CT demonstrated higher diagnostic performance than ¹⁸F-FDG PET/CT and accuracy comparable to breast MRI for primary tumor size estimation, detection of multifocal and multicentric disease, and assessment of axillary lymph node metastasis. While breast MRI remained the most sensitive modality for detecting multifocal and multicentric disease, its specificity for axillary staging was lower. In contrast, FAPI-PET provided a more balanced sensitivity–specificity profile and achieved the highest overall accuracy for axillary evaluation.

These findings suggest that FAPI-PET may serve as a complementary modality to breast MRI, particularly in clinically challenging scenarios where FDG demonstrates limited sensitivity. Larger prospective and subtype-stratified studies are warranted to further define the role of FAPI-PET within multimodal breast cancer staging.

## Data Availability

The datasets generated and analyzed during this study can be available from the correspondence author if reasonably requested.

## Declarations

### Conclusion

⁶⁸Ga-FAPI PET/CT demonstrated superior performance to ¹⁸F-FDG PET/CT and accuracy comparable to MRI for primary tumor size and multifocal and multicentric disease. ⁶⁸Ga-FAPI PET/CT also provided higher specificity than MRI for ALNM and identified additional metastatic sites missed by FDG, supporting its role as a complementary modality to MRI and a promising alternative to FDG-PET/CT in selected patients.
